# Knowledge and practices about zoonotic tuberculosis prevention and associated determinants amongst livestock workers in Nigeria; 2015

**DOI:** 10.1371/journal.pone.0198810

**Published:** 2018-06-11

**Authors:** Hezekiah Kehinde Adesokan, Victor Oluwatoyin Akinseye, Mutolib Abiodun Sulaimon

**Affiliations:** 1 Department of Veterinary Public Health and Preventive Medicine, University of Ibadan, Ibadan, Nigeria; 2 Abakaliki Central Abattoir, Ministry of Agriculture and Natural Resources, Abakaliki, Ebonyi State, Nigeria; Indian Institute of Technology Delhi, INDIA

## Abstract

Zoonotic tuberculosis (TB) is a significant public health disease, but has long been neglected. Information on knowledge and practices of its prevention and influencing factors amongst occupationally exposed individuals is required for designing all-inclusive, informed control programmes. We investigated knowledge and practices related to zoonotic TB prevention and associated determinants amongst herdsmen and abattoir workers in south-western, south-eastern and north-western Nigeria using semi-structured questionnaire. Data were analysed with STATA 12. A total of 510 respondents (196 herdsmen; 314 abattoir workers) participated in the survey, of which 58.6% and 46.9% respectively were knowledgeable and demonstrated good practices about zoonotic TB prevention. Almost 60% knew that zoonotic TB transmission was preventable and 49.8% knew transmission could be through consumption of infected animal products. However, only 16.7% knew the disease could be transmitted by aerosol. Just 49.4% sought medical check-up when ill, 37.8% used protective clothing and only 29.2% usually condemned TB infected cattle. Respondents with post-primary education were about three times more likely to be knowledgeable (OR = 2.70, 95%CI: 1.68–4.33) and two times more likely to demonstrate good practice (OR = 2.23, 95%CI: 1.45–3.42) than those without formal education. Similarly, abattoir workers were about 6.4 times more likely to be knowledgeable (OR: 6.39, 95%CI: 4.31–9.47) and two times more likely to demonstrate good practice (OR: 2.03, 95%CI: 1.40–2.92) than the herdsmen. There were important knowledge gaps with poor practices about zoonotic TB prevention amongst livestock workers in Nigeria. Strong predictors of knowledge and practice were being an abattoir worker and having post-primary education. Well-designed grassroots enlightenment programmes addressing modes of transmission, handling infected cattle and seeking medical check-up are urgently needed among high risk settings considering the recently launched Road Map for Zoonotic Tuberculosis which resonates that every tuberculosis case counts towards 2030 End-TB Strategy.

## Introduction

Tuberculosis (TB) remains a major global health problem [1–2]. It plays a central role in public health and animal health due to its severe disease in humans and significant economic losses to cattle producers related to affected herds and slaughtered cattle [3–6]. It causes ill-health in millions of people each year and in 2015 was one of the top 10 causes of death worldwide, ranking above HIV/AIDS as one of the leading causes of death from an infectious disease [2]. It is caused by *Mycobacterium tuberculosis* in humans resulting in active TB in approximately 10.4 million people in 2015 [2] and *M*. *bovis* in cattle with a broader host range for TB in domestic and wild animals [7]. In addition, *M*. *bovis* infects humans, causing zoonotic TB in humans [8–10]. An estimated 147 000 new cases of zoonotic TB were reported in 2015 globally and 12, 500 deaths due to the disease with the highest incidence in Africa [2]. These global estimates are however imprecise due to lack of routine surveillance data from human and animal populations [11]. For instance, earlier local studies have reported higher proportions in humans [9, 12–13].

The main routes of *M*. *bovis* transmission from infected animal to humans are believed to be through ingestion of raw milk and/or inhalation of aerosol from diseased animal, mainly in settings where pasteurization of milk is not widely established. Despite this, Nigerian communities particularly livestock workers are characterized with risk practices that facilitate zoonotic TB transmission, including consumption of unpasteurized milk, cohabitation with animals, coupled with increasing incidence of immunosuppressive diseases [14]. Unfortunately, this group of occupationally exposed individuals has been grossly neglected and their knowledge as well as preventive practices against zoonotic TB remains poorly investigated. Information on knowledge and practices of zoonotic TB prevention and influencing factors amongst occupationally exposed individuals remains a significant requirement to design all-inclusive, informed grassroots control programmes targeted towards limiting the disease and ultimately achieving the goal of 2030 End-TB strategy. The present study investigated existing levels of knowledge and practice of zoonotic TB prevention and associated determinants amongst herdsmen and abattoir workers in south-western, south-eastern and north-western regions of Nigeria.

## Methods

### Study design, population and selection

**Fig 1:**
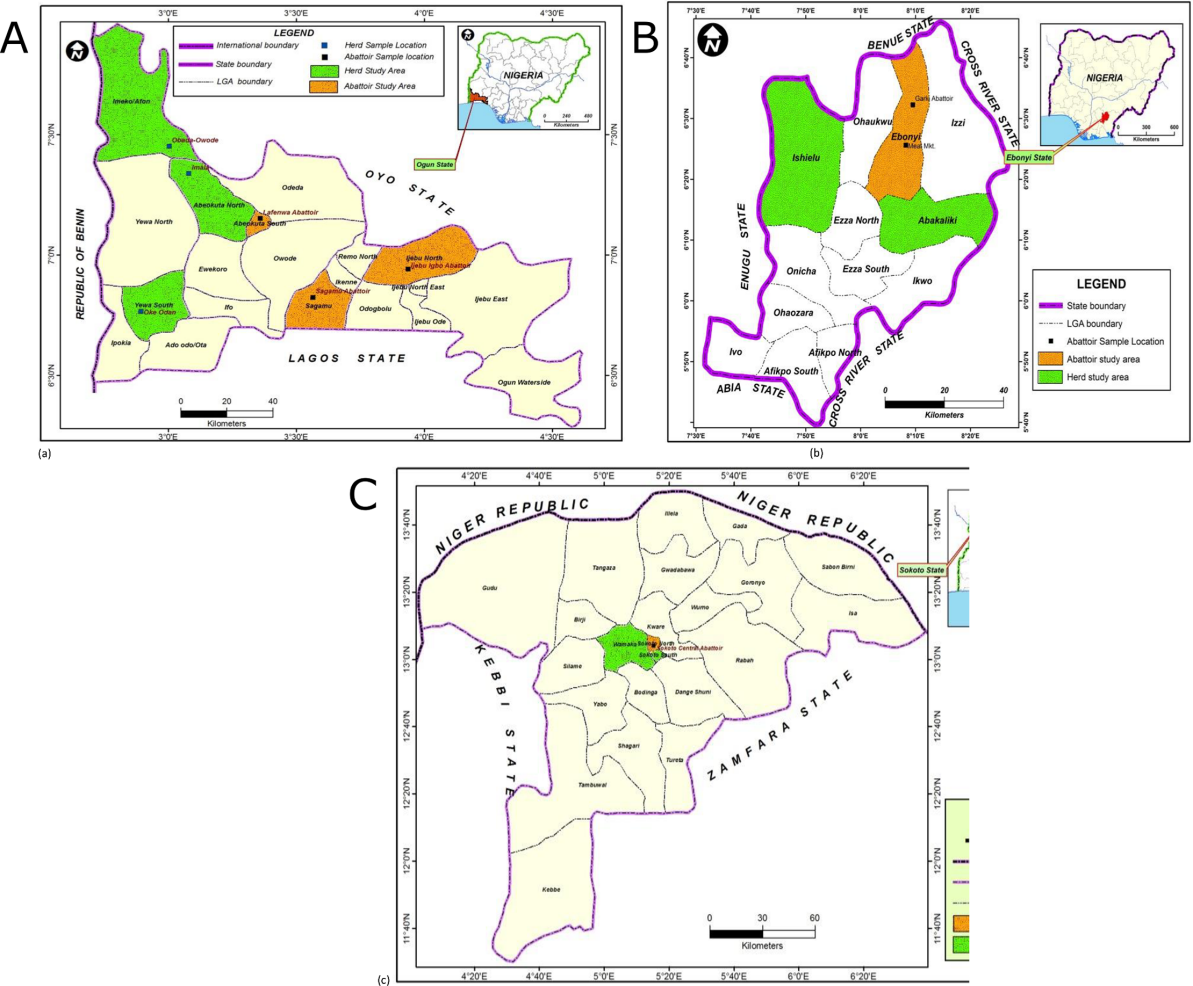
Maps of the three States showing locations where the study was carried out: (a) Ogun (b) Ebonyi (c) Sokoto.

This cross sectional study involved three different states (Ogun, Ebonyi, Sokoto) from three of the six geographical zones in Nigeria, representative of areas with different geographical regions (south-western, south-eastern, north-western, respectively), high cattle production and processing activities (Fig 1A–1C).

These regions are inhabited by about 9.6 million people (6.8% of the Nigerian population based on the 2006 national census [15]) and have major abattoirs sited in their state capitals, with cattle herds domiciled mostly in the rural settings. The herdsmen including the abattoir workers have limited access to good health care facilities and good life styles considering that they are mostly rural dwellers of poor economic and low education status. They spend most of the time with their animals and practice communal life styles, such that they live together within same microenvironments and congregate often in social functions, share almost everything together and often in congested environments. The herdsmen engage in practices that could facilitate zoonotic TB transmission such as consumption of unpasteurized milk, cohabitation with animals and delayed health care seeking. Likewise, abattoir workers process infected animals with bare hands and consume uncooked meat [16]. These practices expose them to the risk of zoonotic TB transmission. We conducted a survey of their knowledge and practices regarding zoonotic TB prevention and associated determinants over a period of six months from April–September, 2015.

Sampling was done through a multistage sampling technique. (i) Abattoir workers’ sampling: Stage One: Three of the six geographical zones in Nigeria were selected using simple random sampling technique by balloting. Stage Two: A state each was selected from the three chosen geographical zones based on high livestock production and processing activities with concomitant potential high risk of human exposure to zoonotic TB infection. Stage three: The major abattoir in each state capital city was purposively selected as it represented the location with highest number of abattoir workers in the states as well as cattle processing activities. Stage four: At each of the abattoirs, the butchers and meat/offal processors who were at least 18 years of age were selected. Exclusion criteria included workers other than butchers and meat/offal processors such as revenue collectors, food hawkers as well as being less than 18 years of age. The purpose of the study was then explained to the potential respondents and they were told that participation was voluntary without any penalty attached for refusal. A pretested semi-structured interviewer administered questionnaire was then administered to approximately 5% of the consenting participants after being randomly selected from consenting participants’ lists.

(ii) Herdsmen’s sampling: A farm settlement-based cross-sectional survey was carried out for the herdsmen. The above first two stages under the abattoir workers’ sampling were also conducted. Stage three: Available herds in each state were identified with the help of the Ministry of Agriculture and Natural Resources in each state; herds that had existed for at least a year were purposively selected. Stage four: The household heads responsible for each of the selected herds were identified. Based on culture, the heads of the family interact first with the society, community, and other visitors and are assumed to have more exposure and knowledge about social and health issues. The purpose of the study was therefore explained to the household heads who then gave oral consent to participate on behalf of the households. Most household heads who culturally gave the initial consent for community participation in the study were elderly people who were largely uneducated.

Households whose heads declined participation as well as wives whose husbands prevented from participating based on culture of not allowing their wives to talk to visitors were exempted from the study. The study participants consisted of consenting heads of herdsmen households and other household members who were at least 18 years of age. A pretested semi-structured interviewer administered questionnaire was then administered to approximately 5% of the consenting participants after being randomly selected from consenting participants’ lists. Considering the settings of study, livestock workers are a homogenous population who are known to have cultural ties, which influence their ways of life and belief. The authors therefore sampled consenting individuals, based on availability, cooperation and accessibility. We believe these responses are generalizable to the populations. In all, 31 farm settlements consisting of 9, 15 and 7 from Ogun, Ebonyi and Sokoto States, respectively participated in the study.

### Data collection

Data were collected using a semi-structured questionnaire. In order to ensure the standardization of the questionnaire, experts from various departments in the University of Ibadan including Veterinary Public Health and Preventive Medicine, Public Health Microbiology unit of the Department of Microbiology, and Social Sciences were consulted. The set of experts and the researchers drafted questions related to zoonotic tuberculosis based on literature. The questions were thereafter reviewed for content validity by the experts and a social scientist. Revisions were made as needed and a pre-test of the questionnaire containing twenty-two questions (10 questions on knowledge and 12 on practices) was conducted prior to the survey on respondents (five abattoir workers and five herdsmen from each of the three regions) who were not part of the study for face validity, including language appropriateness and format. Some of the questions were thereafter revised in order to improve clarity and completeness, but ensuring that the content was still maintained.

The questionnaire (S1–S4 Texts) was first developed in English and then translated into the local language of each region namely Yoruba (Ogun), Igbo (Ebonyi) and Hausa (Sokoto) and back to English by a different individual in order to check for consistency and conceptual equivalence. Participants were interviewed in their respective local languages by trained data collectors. The questionnaire had both open and closed-ended questions and was divided into three parts. The first section consisted of the details of individual characteristics which included age, gender, education level, duration in livestock handling and types of occupation (herdsmen or abattoir workers). Section two covered knowledge of zoonotic tuberculosis prevention, including knowing that zoonotic TB from cattle to humans is preventable, knowledge of modes of transmission and various preventive measures, as well as treatment. The third section focused on practices in relation to zoonotic TB prevention strategies, such as health care seeking practices, actions towards TB infected cattle, preventive practices engaged in, and likely treatment options if infected. Overall, the questionnaire took 20–25 minutes per respondent to fill.

Scores were given according to the accuracy of respondents’ answers, ranging from zero to one per each incorrectly and correctly answered question, respectively. Completely and accurately answered questions attracted overall scores of 10 and 12 for zoonotic TB prevention knowledge and practices, respectively per respondent. A respondent was classified as knowledgeable if he scored six or more of the knowledge questions, and good practices if he scored eight or more of the practice questions which is equal to or more than 60%.

### Statistical analysis

Data were analysed using STATA 12. Frequencies and percentages were calculated as appropriate for knowledge and practices of zoonotic TB prevention. Univariate binary logistic analysis and multivariate logistic regression analysis were conducted to determine the relationships between outcome variables (of whether respondents were knowledgeable about zoonotic TB prevention or not and whether they demonstrated good practice or not) and a range of factors (such as age, gender, education level, duration and occupation types). Odds ratios (OR) were computed to determine the presence and strength of the associations between variables and 95% Confidence Intervals (CIs) were calculated to investigate statistical significance for each predictor variable.

### Ethics statement

The ethical clearance for the study protocols was obtained from the University of Ibadan/University College Hospital Institutional Review Board with the approval number NHREC/05/01/2008a. All potential participants were adults given the exclusion criterion of not including ages below 18 years in the study. The purpose and benefits of the study were explained to them. They were told that participation was voluntary. Oral consent was obtained from each participant considering the fact that many of them could not read or write. The Confidentiality of the respondents was maintained by using number codes on the questionnaire without any use of individual names. Oral consents obtained were documented in the project notebook against the respective codes on the questionnaire administered to each respondent. The use of oral consents in such settings as this is in line with the institutional ethics committee of the University and was approved by them.

## Results

### Socio-demographic characteristics of respondents

A total of 510 livestock workers comprising 314 (61.6%) abattoir workers and 196 (38.4%) herdsmen participated in the study. The highest proportion (41.0%) of the respondents was within the age group 40 years and above, 64.9% were males and 36.9% had post-primary education. The majority of the respondents (59.0%) had been dealing in livestock business for over three years (Table 1).

**Table 1:** Socio-demographic characteristics of respondents in Nigeria (N = 510).

Variable	Category	Frequency	Percentage
**Age (in years)**	18–29	151	29.6
	30–39	150	29.4
	≥ 40	209	41.0
**Gender**	Male	331	64.9
	Female	179	35.1
**Education level**	None	164	32.1
	Primary	158	31.0
	Post-primary	188	36.9
**Duration** **(in years)**	<1	79	15.5
	1–3	130	25.5
	>3	301	59.0
**Occupation types**	Herdsmen	196	38.4
	Abattoir workers	314	61.6

### Zoonotic tuberculosis infection prevention knowledge and determinants

Almost 60% of the respondents were knowledgeable about zoonotic TB prevention. The knowledge level was higher among the abattoir workers (75.5%) than the herdsmen (32.1%). On zoonotic TB prevention related questions, 58.0% of the respondents knew that TB transmission from cattle to man was preventable. Only 49.8% and 16.7% indicated consumption of infected animal products (unpasteurized milk and meat) and inhalation through direct contacts as modes of transmission, respectively, while 37.5% did not know the mode of transmission. In addition, 62.9% knew that zoonotic TB can be cured. However, only 55.1% knew modern medicine as the best form of treatment; others indicated traditional medicine (28.7%) and prayer (16.2%).

Multivariate logistic regression models indicated that only the gender, education level and occupation types were strongly associated with zoonotic TB prevention knowledge. The female respondents were about twice less likely to be knowledgeable about zoonotic TB prevention than the males (OR = 0.49, 95%CI: 0.33–0.74). Respondents with post-primary education were about three times more likely to be knowledgeable than those without formal education (OR = 2.70, 95%CI: 1.68–4.33). The abattoir workers were about 6.4 times more likely to be knowledgeable about zoonotic TB prevention than the herdsmen (OR: 6.4, 95%CI: 4.31–9.47) (Table 2; S1 and S2 Tables).

**Table 2:** Factors influencing levels of knowledge about zoonotic TB prevention amongst livestock workers in Nigeria (N = 510).

Variable	Category	Knowledgeable n (%)	Not knowledgeable n (%)	OR, 95%CI, P value
**Age** **(in years)**	18–29	94 (62.3)	57 (37.7)	1.00 (reference)
	30–39	65 (43.3)	85 (56.7)	0.41, 0.25–0.68, 0.001
	≥ 40	140 (67.0)	69 (33.0)	0.97, 0.59–1.57, 0.885
**Gender**	Male	214 (64.7)	117 (35.3)	1.00 (reference)
	Female	85 (47.5)	94 (52.5)	0.49, 0.33–0.74, 0.001
**Education level**	None	76 (46.3)	88 (53.7)	1.00 (reference)
	Primary	91 (57.6)	67 (42.4)	2.26, 1.38–3.70, 0.001
	Post-primary	132 (70.2)	56 (29.8)	2.70, 1.68–4.33, 0.000
**Duration** **(in years)**	<1	40 (50.6)	39 (49.4)	1.00 (reference)
	1–3	54 (41.5)	76 (58.5)	0.56, 0.30–1.05, 0.071
	>3	205 (68.1)	96 (31.9)	1.70, 0.99–2.94, 0.056
**Occupation types**	Herdsmen	63 (32.1)	133 (67.9)	1.00 (reference)
	Abattoir workers	236 (75.1)	78 (24.9)	6.39, 4.31–9.47, 0.000

### Zoonotic tuberculosis infection prevention practices and determinants

Regarding zoonotic TB prevention practices among the respondents, less than half (46.9%) demonstrated good practice of zoonotic TB prevention. A significantly higher proportion of the abattoir workers (53.5%) demonstrated good practice of zoonotic TB prevention than the herdsmen (36.2%). Just over half (58.2%) of the respondents had history and evidence of BCG vaccination, 25.7% practised self-medication while 6.1% relied on the use of herbs and 10.0% on prayer in order to prevent zoonotic TB infection. Responding to how they handled TB infected cattle, 42.2% usually sold them to the public, 29.2% slaughtered and buried, 10.8% usually slaughtered them for their home consumption while 17.8% were indifferent. Again, the majority (71.4%) of the respondents indicated that they would seek hospital treatment in case they discovered that they are infected with TB. However, 20.6% said they would use traditional medicine while 8.0% would seek spiritual solution. In addition, only 49.4% sought medical check-up. On the type of hygiene practices they engaged in to prevent zoonotic TB transmission, 37.8% put on protective clothing while working with animals, 22.7% indicated ensuring not touching animals or carcasses with bare hands while 13.5% always washed their hands after touching live or processed animal carcass. However, 25.9% did not engage in any form of preventive practices against zoonotic TB transmission.

Multivariate regression models revealed that education level, duration and occupation types were the strong determinants of the respondents’ zoonotic TB prevention practices. Respondents with post-primary education were about two times more likely to demonstrate good practices (OR = 2.23, 95%CI: 1.45–3.42) than those without formal education. In addition, respondents who had spent more than three years in livestock business were about twice more likely to demonstrate good practices than those who had spent less than 3 years (OR = 2.34, 95%CI: 1.39–3.92) The abattoir workers were two times more likely to demonstrate good practices of zoonotic TB prevention than the herdsmen (OR = 2.03; 95%CI: 1.40–2.92) (Table 3; S3 and S4 Tables).

**Table 3:** Factors influencing levels of practices about zoonotic TB prevention amongst livestock workers in Nigeria (N = 510).

Variable	Category	Good practice n (%)	Poor practice n (%)	OR, 95%CI, P value
**Age**	18–29	79 (52.3)	72 (47.7)	1.00 (reference)
	30–39	49 (32.7)	101 (67.3)	0.44, 0.27–0.71, 0.001
	≥ 40	111 (53.1)	98 (46.9)	1.03, 0.68–1.57, 0.882
**Gender**	Male	166 (50.2)	165 (49.8)	1.00 (reference)
	Female	73 (40.8)	106 (59.2)	0.69, 0.47–0.99, 0.043
**Education level**	None	67 (40.9)	97 (59.1)	1.00 (reference)
	Primary	58 (36.7)	100 (63.3)	0.84, 0.54–1.32, 0.446
	Post-primary	114 (60.6)	74 (39.4)	2.23, 1.45–3.42, 0.000
**Duration** **(in years)**	<1	27 (34.2)	52 (65.8)	1.00 (reference)
	1–3	47 (36.2)	83 (63.8)	1.09, 0.61–1.96, 0.772
	>3	165 (54.8)	136 (45.2)	2.34, 1.39–3.92, 0.001
**Occupation types**	Herdsmen	71 (36.2)	125 (63.8)	1.00 (reference)
	Abattoir workers	168 (53.5)	146 (46.5)	2.03, 1.40–2.92, 0.000

## Discussion

Adequate knowledge and effective practices of zoonotic TB prevention among the occupationally exposed groups particularly in sub-Saharan African countries where the burden of TB is high is vital to achieve the WHO’s 2030 End TB Strategy which seeks to end the global TB epidemic by 2030. This becomes more important in the wake of the recently launched Road Map for Zoonotic Tuberculosis by WHO/OIE/FAO/IUATLD [11] and supported by the Stop TB Partnership’s Global Plan to End TB 2016–2020 –The Paradigm Shift, which identifies people at risk of zoonotic TB as a neglected population deserving greater attention [11].

The current study reports on the level of knowledge and practices of zoonotic TB prevention among livestock workers (abattoir workers and herdsmen) in Nigeria. The study revealed that though the livestock workers were knowledgeable about zoonotic TB prevention, there were knowledge gaps in some important areas. For instance, more than half and the majority, respectively did not know consumption of unpasteurized milk or meat from infected animals and inhalation by direct contact as modes of transmission of zoonotic TB. This finding is disturbing considering the exposure risks associated with such a high risk group especially given the practice of consumption of unpasteurized milk and uncooked meat earlier reported to be common among them [16, 17–18]. Meanwhile, various reports have established the presence of *M*. *bovis* in milk and meat from infected animals in Nigeria and elsewhere [19–22]. Likewise, Adesokan *et al*. [9] reported the isolation of *M*. *bovis* strain previously documented in cattle among livestock workers in south-western Nigeria. While public health significance of consumption of *M*. *bovis*-infected meat has not been clearly established, the growing habit of consuming raw uncooked meat [16, 23] particularly in Africa where food safety hygiene is poor is a matter of concern. More so, in most countries in Africa where bovine TB is prevalent; effective disease control, including regular milk pasteurization and slaughterhouse meat inspection, is largely absent [3, 8]. This situation is exacerbated by the presence of other additional risk factors such as human behavior and the high prevalence of HIV infections [3, 24]. Some studies showed a significantly increased proportion of *M*. *bovis* infections among HIV–co-infected TB patients compared with HIV-negative TB patients [25–27]. The current finding of poor knowledge of mode of transmission of zoonotic TB among livestock workers in the study areas is an important knowledge gap of concern and portends serious challenge to the control of TB, thus requires urgent attention.

Despite the overall knowledge level about zoonotic TB prevention observed among the respondents in this study, only less than half demonstrated good practices. The observations that less than half sought medical check-ups and more than one-thirds would either depend on self-medication, use herbs or prayer to prevent or treat zoonotic TB infection in this study suggest the need for more enlightenment campaigns regarding prevention of zoonoses among the occupationally exposed groups. As also observed, almost one third of the respondents would seek non-modern medical approach including the use of herbs if infected with zoonotic TB. This finding might explain why previous reports showed that livestock workers in developing countries exhibit poor health care-seeking behaviours [9, 33]. Such poor practices might in turn, result in prolonged diagnostic and treatment delay thereby facilitating the spread of the disease among the livestock worker community. It becomes very imperative that measures towards limiting these exposure risk practices among livestock workers generally in developing countries are put in place.

The study also shows that the abattoir workers were more than six times knowledgeable and two times more likely to demonstrate good practices about zoonotic TB prevention than the herdsmen. This finding is of significant epidemiological implications for zoonotic TB control. Available reports show that TB spreads primarily by the aerogenic pathway among cattle, and that those directly in contact with them are more likely to develop pulmonary disease than an alimentary form [28–29]. The herdsmen are therefore at higher risk of infection with zoonotic TB than the abattoir workers given the longer contact time with their live animals. More so, it requires only minute quantity of bacilli to establish infection in humans through aerogenous route to which herdsmen are more exposed compared with higher doses through ingestion [30]. Our findings are in agreement with previous reports [16, 31–32] which indicated lower knowledge level of zoonotic TB among high risk group.

Further, our findings reveal that respondents with post-primary education were about three and two times, respectively more likely to be knowledgeable and demonstrate good practices about zoonotic TB prevention than those without formal education. Similar reports have been made in Edo and Zamfara States, Nigeria [34–35], Tanzania [36] as well as China [37], showing that education contributes significantly to knowledge and practices regarding TB. Unfortunately, most livestock workers are often illiterate because their access to education is limited [38–39] since they spend most of their lives in remote rural areas. Consequently, they are rarely considered by national intervention and development activities [4]. There is a need to strengthen the existing global TB control programmes to include the livestock workers regarded as neglected people in the fight against TB [2] by promoting an extensive grassroots health education programme to raise their awareness specifically about TB symptoms, means of transmission, prevention, and treatment. Public health education training programmes should also be provided to members of the livestock communities considering the cultural ties and communal lifestyles which often characterize this setting.

This study had some limitations. One, active case detection for the presence of zoonotic TB was not conducted as this would have shown the magnitude of the disease burden among the livestock workers studied. However, previous studies have established the presence of zoonotic TB among livestock workers in Nigeria [9, 40]. Two, only three states were selected for the study. These three states represent three of the six geographical zones in Nigeria and are known for increased cattle production and processing. More so, livestock workers have cultural ties which make their beliefs and practices similar across the country. Hence, the findings in this study could generalize the situations across the states in Nigeria.

## Conclusion

The livestock workers were knowledgeable about zoonotic TB prevention; however, there were important knowledge gaps in some core areas, including its modes of transmission coupled with their poor levels of preventive practices. Education and types of occupation were significant factors for knowledge and practices about zoonotic tuberculosis prevention amongst the livestock workers. Considering the fact that the greatest burden of zoonotic diseases including tuberculosis lies within poor, marginalised, rural communities that live in close proximity with livestock and lack access to safe food and adequate health care, there is need to step up increased awareness programmes about these diseases amongst such settings. Since most zoonotic diseases often share common risk factors, well-designed education prevention strategies can reduce risks posed by several diseases at once, thereby increasing their overall cost- and resource-effectiveness. Hence, grassroots enlightenment programmes on zoonotic disease preventive measures are urgently needed amongst livestock communities especially in developing countries in line with the goal of the recently launched Road Map for Zoonotic Tuberculosis by WHO/OIE/FAO/IUATLD [11] towards achieving 2030 End-TB Strategy.

## Supporting information

S1 Text Questionnaire on knowledge and practice of livestock workers about zoonotic tuberculosis prevention (English). (DOC)

S2 Text Questionnaire on knowledge and practice of livestock workers about zoonotic tuberculosis prevention (Yoruba). (DOC)

S3 Text Questionnaire on knowledge and practice of livestock workers about zoonotic tuberculosis prevention (Igbo). (DOCX)

S4 Text Questionnaire on knowledge and practice of livestock workers about zoonotic tuberculosis prevention (Hausa). (DOCX)

S1 Table Raw data on knowledge assessment of livestock workers on zoonotic TB prevention. (SAV)

S2 Table Assessment scores of the knowledge levels of livestock workers on zoonotic TB prevention. (XLS)

S3 Table Raw data on assessment of practices of livestock workers on zoonotic TB prevention. (SAV)

S4 Table Assessment scores of the preventive practice levels of livestock workers against zoonotic TB. (XLS)
