# Supplementary material for: Knowledge and practices about zoonotic tuberculosis prevention and associated determinants amongst livestock workers in Nigeria; 2015
Source: PLoS One. 2018 Jun 11;13(6):e0198810. doi: 10.1371/journal.pone.0198810 (PMC5995405; doi:10.1371/journal.pone.0198810)
Supplement: S1 Text — (DOC) [file pone.0198810.s001.doc]

**QIN UI/*/TB/2015***

**Knowledge and Practice of Livestock Workers about zoonotic tuberculosis prevention**

**Site:_______________**

**Knowledge and Practice of Livestock Workers about zoonotic tuberculosis (TB) prevention**

**SECTION A: DEMOGRAPHIC AND SOCIOECONOMIC CHARACTERISTICS OF RESPONDENTS**

| QN | QUESTIONS & FILTERS | RESPONSE OPTIONS | CODE | Skip to |
| --- | --- | --- | --- | --- |
| A1 | Occupation type | Abattoir workers……………………………  Herdsmen………………………………….. | 1  2 |  |
| A2 | How old were you as at your last birthday? | 18 -29………………………………………  30 – 39……………………………………..  40 – 49 …………………………………….  50 – 59 …………………………………….  60 and above ……………………………..  - | 1  2  3  4  5 |  |
| A3 | Record sex of the respondent | Male ----------------------------------------------  Female------------------------------------------- | 1  2 |  |
| A4 | What is your highest level of educational attainment? | No formal education --------------------------  Primary -------------------------------------------  Secondary ---------------------------------------  Tertiary ------------------------------------------  Others (specify) __________________ | 1  2  3  4  5 |  |
| A5 | How long have you been in this business? | Less than one year ………………………  1 – 3 years ……………………………….  More than 3 years ………………………. | 1  2  3 |  |

**SECTION B: KNOWLEDGE OF ZOONOTIC TB PREVENTION**

| QN | QUESTIONS & FILTERS | RESPONSE OPTIONS | CODE | SKIP TO |
| --- | --- | --- | --- | --- |
| B1 | What are the clinical signs of TB in animals? (List at least two) |  |  |  |
| B2 | Does zoonotic tuberculosis affect humans? | Yes………………………………………..  No…………………………………………  I don’t know……………………………… | 1  2  3 |  |
| B3 | Zoonotic TB could be transmitted through which means? | Consumption of infected animal products (unpasteurized milk and meat)…………  Inhalation…………………………………  I don’t know………………………………  Others (Specify)…………………………. | 1  2  3  4 |  |
| B4 | What are the symptoms of TB infection in humans?  (List at least two) |  |  |  |
| B5 | Is zoonotic TB from cattle to man preventable? | Yes………………………………………..  No…………………………………………  I don’t know……………………………… | 1  2  3 |  |
| B6 | Boiling milk before drinking kills the bacteria organism causing zoonotic TB | Yes………………………………………..  No…………………………………………  I don’t know……………………………… | 1  2  3 |  |
| B7 | Separating animal pens from that of humans reduces infection with zoonotic TB | Yes………………………………………..  No…………………………………………  I don’t know……………………………… | 1  2  3 |  |
| B8 | Zoonotic TB in man is curable | Yes………………………………………..  No…………………………………………  I don’t know……………………………… | 1  2  3 |  |
| B9 | What type of treatment do you think is best for TB? | Modern medicine……………………………  Traditional medicine………..………………..  Prayer………………………………………  Others (Specify)…………………………….. | 1  2  3  4 |  |
| B10 | TB treatment in man is free | Yes………………………………………..  No…………………………………………  I don’t know……………………………… | 1  2  3 |  |

**SECTION C: PRACTICES ABOUT ZOONOTIC TB PREVENTION**

| QN | QUESTIONS & FILTERS | RESPONSE OPTIONS | CODE | SKIP TO |
| --- | --- | --- | --- | --- |
| C1 | How do you prevent being infected with TB from cattle? | BCG vaccination (Researcher checks to confirm)……………………………………….  Practise self-medication…………………….  Use herbs…………………………………….  Prayer………………………………………… | 1  2  3  4 |  |
| C2 | I do not drink unpasteurized milk | Yes………………………………………  No………………………………………. | 1  2 |  |
| C3 | What do you do when your cattle has TB? | Sell to the public…………………………  Slaughter and bury it……………………  Slaughter for home consumption……..  Others (specify)………………………. | 1  2  3  4 |  |
| C4 | I always notify veterinarians in case of infection in cattle or carcass | Yes………………………………………  No………………………………………. | 1  2 |  |
| C5 | I always allow inspection of my animals by veterinarians | Yes………………………………………  No………………………………………. | 1  2 |  |
| C6 | I always pack animal dungs with my hands gloved | Yes………………………………………  No………………………………………. | 1  2 |  |
| C7 | I do not co-habit with animals in my house | Yes………………………………………  No………………………………………. | 1  2 |  |
| C8 | What would you do if you are infected with zoonotic TB? | Seek treatment in the hospital……………  Use traditional medicine…………………..  Seek spiritual solution……………………. | 1  2  3 |  |
| C9 | I visit hospital for regular medical check-ups | Yes………………………………………  No………………………………………. | 1  2 |  |
| C10 | I always protect my wounds while handling or processing animals | Yes………………………………………  No………………………………………. | 1  2 |  |
| C11 | I do not eat while handling or processing animals | Yes………………………………………  No………………………………………. | 1  2 |  |
| C12 | What are your hygiene practices against zoonotic TB transmission? List them |  |  |  |
