# Supplementary material for: Knowledge and practices about zoonotic tuberculosis prevention and associated determinants amongst livestock workers in Nigeria; 2015
Source: PLoS One. 2018 Jun 11;13(6):e0198810. doi: 10.1371/journal.pone.0198810 (PMC5995405; doi:10.1371/journal.pone.0198810)
Supplement: S2 Text — (DOC) [file pone.0198810.s002.doc]

**NOMBA UI/*/IKO FEE/2015***

**Imo ati ise awon to n sise pelu eran osin nipa dideena iko fee to n ti ara eranko bo si ara eniyan**

**Ibi Ise:_______________**

**Imo ati ise awon to n sise pelu eran osin nipa dideena iko fee to n ti ara eranko bo si ara eniyan**

**IPIN A: KOKO AJEMENI LAWUJO**

| NOMBA | AWON IBEERE | AWON EDA IDAHUN | NOMBA TO DURO FUN IDAHUN |
| --- | --- | --- | --- |
| A1 | Irufe Ise ti e n se | Osise Odo Eran ……………………………  Darandaran………………………………… | 1  2 |
| A2 | Ojo ori re | Mejidinlogun –Ookandinlogbon….………  Ogbon – Ookandinlogoji…………………..  Ogoji – Ookandinlaadota………………….  Aadota – Ookandinlogota..……………….  Ogota ati ju bee lo…..……………………..  - | 1  2  3  4  5 |
| A3 | Okunrin tabi Obinrin | Okunrin--------------------------------------------  Obinrin ------------------------------------------- | 1  2 |
| A4 | Iwe eri ile iwe | N o lo si ile-iwe moon-ko moon-ka----------  Ile-iwe Oniwe mefa-----------------------------  Ile-iwe Oniwe mewa----------------------------  Ile-iwe giga---------------------------------------  Omiiran (so o ni paato)____________ | 1  2  3  4  5 |
| A5 | O ti to igba wo ti o ti wa lenu ise yii? | Ko to odun kan ………………………  Odun kn si meta……………………….  O ti ju odun meta lo………………………. | 1  2  3 |

**IPIN B: IMO NIPA DIDEENA IKO FEE TI O N TI ARA ERANKO BO SI ARA ENIYAN**

| NOMBA | AWON IBEERE | AWON EDA IDAHUN | NOMBA TO DURO FUN IDAHUN |
| --- | --- | --- | --- |
| B1 | Kinni awon aami to n safihan iko fee ninu eranko? (Daruko o kere ju meji ninu won) |  |  |
| B2 | Nje iko fee eranko lee mu eniyan? | Beeni...……………………………………..  Beeko………………………………………  N o mo…….……………………………… | 1  2  3 |
| B3 | Ona wo ni iko fee eranko lee gba dara eniyan? | Jije eran tabi mimu wara to ni kokoro iko fee……………………………………………  Nipa se eemi…………………………………  N o mo ………………………………  Omiiran (so o ni paato……………………. | 1  2  3  4 |
| B4 | Kinni awon aami to n safihan iko fee ninu eniyan? (Daruko o kere ju meji ninu won) |  |  |
| B5 | Nje iko fee lati ara maalu si eniyan see deena? | Beeni...……………………………………..  Beeko………………………………………  N o mo…….……………………………… | 1  2  3 |
| B6 | Sise wara ki a to o mu u yoo pa kokoro to n fa iko fee to ran eniyan lati ara eranko | Beeni...……………………………………..  Beeko………………………………………  N o mo…….……………………………… | 1  2  3 |
| B7 | Pipala saarin ile eran ati ibugbe eniyan lee dekun iko fee to ran eniyan lati ara eranko | Beeni...……………………………………..  Beeko………………………………………  N o mo…….……………………………… | 1  2  3 |
| B8 | Iko fee to n ran eniyan lati ara eranko gboogun | Beeni...……………………………………..  Beeko………………………………………  N o mo…….…………… ………………… | 1  2  3 |
| B9 | Irufe itoju wo lo lero pe o dara julo fun iko fee? | Isegun igbalode……………………………  Isegun ibile………………..………………..  Adura………………………………………  Omiiran (so o ni paato…………….……….. | 1  2  3  4 |
| B10 | Ofe ni itoju iko fee fun eniyan | Beeni...……………………………………..  Beeko………………………………………  N o mo…….……………………………… | 1  2  3 |

**IPIN D: ISESI NIPA DIDEENA IKO FEE TO N TI ARA ERANKO BO SI ARA ENIYAN**

| NOMBA | AWON IBEERE | AWON EDA IDAHUN | NOMBA TO DURO FUN IDAHUN |
| --- | --- | --- | --- |
| D1 | Bawo ni o n se deena kiko iko fee lati ara maalu? | Gbigba abeere ajesera to n deena iko fee (oluwadii se ayewo fun ifidi I re mu le)…..  Lilo ogun atowo da ara eni………………….  Lilo ewe ati egbo…………………………….  Gbigba adura………………………………… | 1  2  3  4 |
| D2 | N o ki i mu wara ti a ko se | Beeni………………………………………  Beeko………………………………………. | 1  2 |
| D3 | Kinni ohun ti o ma n se nigba ti maalu re ba ni iko fee? | Ta a sita…………………………  Mo ma n pa ki n si sin mole………………  Pa a fun jije ni ile………………………..…..  Omiiran (so o ni paato)………………. | 1  2  3  4 |
| D4 | Mo ma n fi to awon dokita eranko leti nigba ti eran mi ba ni arun kan tabi omiiran | Beeni………………………………………  Beeko..……………………………………. | 1  2 |
| D5 | Mo ma n gba awon dokita eranko lati se ayewo awon eran mi | Beeni………………………………………  Beeko..……………………………………. | 1  2 |
| D6 | Mo ma n wo ibowo nigba ti mo ba n ko igbe eran | Beeni………………………………………  Beeko..……………………………………. | 1  2 |
| D7 | N o jo gbe pelu eran ninu ile igbe mi | Beeni………………………………………  Beeko..……………………………………. | 1  2 |
| D8 | Kinni iwo yoo se ti o ba ni iko fee lati ara eran? | Lo fun itoju nile isegun igbalode……………  Lo ogun ibile………………...………………..  Wa ona abayo ti emi…..……………………. | 1  2  3 |
| D9 | Mo ma n lo si ile isegun loorekoore fun ayewo | Beeni………………………………………  Beeko..……………………………………. | 1  2 |
| D10 | Mo ma daabo bo egbo mi nigba ti mo ba n sise pelu eran | Beeni………………………………………  Beeko..……………………………………. | 1  2 |
| D11 | N o ki jeun nigba ti mo ba n sise pelu eran | Beeni………………………………………  Beeko..……………………………………. | 1  2 |
| D12 | Kinni awon isesi imototo ti o ma n se lati deena iko fee lati ara eranko? Daruko won |  |  |
