# Supplementary material for: Knowledge and practices about zoonotic tuberculosis prevention and associated determinants amongst livestock workers in Nigeria; 2015
Source: PLoS One. 2018 Jun 11;13(6):e0198810. doi: 10.1371/journal.pone.0198810 (PMC5995405; doi:10.1371/journal.pone.0198810)
Supplement: S3 Text — (DOCX) [file pone.0198810.s003.docx]

**QIN UI/*/TB/2015***

**Omuma na omume ndi na-achi ma obu na-egbu ehi maka mgbochi oria ukwara nta si na anu abanye na mmadu**

**Ebe ano:_______________**

**Omuma na omume ndi na achi ehi maka mgbochi oria ukwara nta si na anu abanye na mmadu**

**AGBA A: UDIRI NA AKPA NDI MMADU AJURU AJUJU**

| USORO AJUJU | AJUJU DI MKPA | AZIZA NA USORO | AKARA | MAFERE |
| --- | --- | --- | --- | --- |
| A1 | Udi aka oru | Ogbu anu……………………………  Ochi ehi………………………………….. | 1  2 |  |
| A2 | Afo ole ka idi ugbua? | Iri na asato ruo iri abuo na itolu…………  Iri ato ruo iri ato na itolu………………..  Iri ano ruo irir ano na itolu……………….  Iri ise ruo iri ise na itolu………………….  Iri isii na karia …………………………….. | 1  2  3  4  5 |  |
| A3 | Nwoke ma obu nwanyi | Nwoke--------------------------------------------  Nwanyi------------------------------------------- | 1  2 |  |
| A4 | Kedu akwukwo ikpeazu igara ruo na isi? | Agaghi ma oli ---------------------------------  Elementiri----------------------------------------  Koloji------- --------------------------------------  Ulo akwukwo di elu---------------------------  Ndi ozo (kowaa) _________________ | 1  2  3  4  5 |  |
| A5 | Afo ole ka ino na orua? | Pekariri otu afo ………………………  Otu afo ruo ato…………………………….  Kariri afo ato ………………………. | 1  2  3 |  |

**Agba B: OMUMA MAKA ORIA NTA SI NA ANU ABANYE NA MMADU**

| USORO AJUJU | AJUJU DI MKPA | AZIZA NA USORO | AKARA | MAFERE |
| --- | --- | --- | --- | --- |
| B1 | Kedu akara eji ama oria ukwara nta? (Deputa opekata mpe abua) |  |  |  |
| B2 | Oria ukwara nta, osi na anu abanye mmadu? | Eee…………………………………………..  Mba.…………………………………………  Amaghi m…..……………………………… | 1  2  3 |  |
| B3 | Kedu uzo ukwara nta si esi na anu abanye mmadu? | Iri ihe oriri sin a anu puta (dika mmiri ara ehi ma obu anu)………………….………  Ikuru ume…………………………………  Amaghi m………………………………  Ndi ozo (kowaa)…………………………. | 1  2  3  4 |  |
| B4 | Kedu mgbu imara na-egbu onye oria ukwara nta?  (Deputa opekata mpe abua) |  |  |  |
| B5 | Ukwara nta si na anu onwere mgbochi? | Eee…..………………………………………..  Mba……………………………………………  Amaghi m …………………………………… | 1  2  3 |  |
| B6 | Isi mmiri ara ehi na oku tupu anuo na-egbochi ibuta ukwara nta site na anu | Eee…..………………………………………..  Mba……………………………………………  Amaghi m …………………………………… | 1  2  3 |  |
| B7 | Ikeweputa ebe obibi mmadu na nke anu ga ebelata mbufe oria ukwara nta si na-anu | Eee…..………………………………………..  Mba……………………………………………  Amaghi m …………………………………… | 1  2  3 |  |
| B8 | A na agwo ukwara nta si na-anu agwo | Eee…..………………………………………..  Mba……………………………………………  Amaghi m …………………………………… | 1  2  3 |  |
| B9 | Kedu etu kasi mma esi agwo ukwara nta si na-anu? | Dibia oyibo……………………………………  Dibia Igbo…………..……..………………..  Epere….………………………………………  Ndi ozo (kowaa)…………………………….. | 1  2  3  4 |  |
| B10 | Igwo ukwara nta bu na nkiti | Eee…..………………………………………..  Mba……………………………………………  Amaghi m …………………………………… | 1  2  3 |  |

**Agba CH: OMUME NA EGBOCHI UKWARA NTA SI NA ANU**

| USORO AJUJU | AJUJU DI MKPA | AZIZA NA USORO | AKARA | MAFERE |
| --- | --- | --- | --- | --- |
| CH1 | Kedu ka isi egboch onwegi ibuta oria ukwara nta si na ehi? | Igba ogu mgbochi BCG (Lele ma ogbara ya)…………………………………………….  Ana m azuru ogu agwo onwe m………..….  Eji m mkporogu na mkpa akwukwo……….  Ekpere…………………………….………… | 1  2  3  4 |  |
| CH2 | Adighi m anu mmiri ara ehi esighi na oku | Eee…..……………………………………  Mba………………………………………. | 1  2 |  |
| CH3 | Kedu ihe I na-eme mgbe ichoputara na ehi gi nwere ukwara nta? | Ree ya n’ ahia………………….…………  Gbuo ma lie ya na-ala ……………………  Gbuo ma rie ya na be m……..…….……..  Ndi ozo (kowaa)………………..…………. | 1  2  3  4 |  |
| CH4 | Ana m akpo dokita anu mgbe obula m choputara ukwara nta na ehi m mgbe odi ndu ma obu mgbe onwuru anwu | Eee..………………………………………  Mba………………………………………. | 1  2 |  |
| CH5 | Ana m ahapu dokito anu ka olelee anu m nke oma | Eee…..……………………………………  Mba………………………………………. | 1  2 |  |
| CH6 | Ana m eyi akpa na aka mgbe obula m choro ikpochapu nsi ehi m | Eee..………………………………………  Mba………………………………………. | 1  2 |  |
| CH7 | Umu anu m nwere ulo nke ha iche na ebe m bi | Eee….……………………………………  Mba………………………………………. | 1  2 |  |
| CH8 | Kedu ihe iga eme ma ichoputa na inwere ukwara nta esi na anu ebuta? | Gaa ulo ogu ga nara ogu……..……………  Gaa na nke dibia Igbo nara ogu …………..  Gaa ulo uka ekpere…………………………. | 1  2  3 |  |
| CH9 | Ana m aga ulo ogu oge obula iIe ka ahu di m | Eee………………………………………  Mba………………………………………. | 1  2 |  |
| CH10 | A na m emechi onya obula di m na ahu mgbe obula m na emetu anu aka ma obu gbuo anu | Eee.………………………………………  Mba………………………………………. | 1  2 |  |
| CH11 | A dighi m eri ihe obula mgbe obula m na emetu ma obu egbu anu | Eee………………………………………  Mba………………………………………. | 1  2 |  |
| CH12 | Kedu ka isi edebe onwe gi ocha maka mgbochi ukwara nta si na anu? (Guputa ha) |  |  |  |
