# Supplementary material for: Knowledge and practices about zoonotic tuberculosis prevention and associated determinants amongst livestock workers in Nigeria; 2015
Source: PLoS One. 2018 Jun 11;13(6):e0198810. doi: 10.1371/journal.pone.0198810 (PMC5995405; doi:10.1371/journal.pone.0198810)
Supplement: S4 Text — (DOCX) [file pone.0198810.s004.docx]

**QIN UI/*/TB/2015***

**Masaniya da Ayukan Ma’aikatan Dabbobi akan tsaron cutar tarin fuka mai yaduwa ga dabbobi da mutane**

**Wurin da ka ke/ki ke:_______________**

**Masaniya da Ayukan Ma’aikatan Dabbobi akan tsaron cutar tarin fuka mai yaduwa gadabbobi da mutane**

**SECTION A: DEMOGRAPHIC AND SOCIOECONOMIC CHARACTERISTICS OF RESPONDENTS**

| QN | QUESTIONS & FILTERS | RESPONSE OPTIONS | CODE | Skip to |
| --- | --- | --- | --- | --- |
| A1 | Irin Sana’ar ka/ki | Mahauta……………………………  Makiyayi………………………………….. | 1  2 |  |
| A2 | Shekarun ka/ki nawa ne? | 18 -29………………………………………  30 – 39……………………………………..  40 – 49 …………………………………….  50 – 59 …………………………………….  60 zuwa sama ………………………….. | 1  2  3  4  5 |  |
| A3 | Mace ne ko na miji? | Na miji----------------------------------------------  Na mace------------------------------------------- | 1  2 |  |
| A4 | Menene matsayin iliminka? | Ba karatun boko ------------------------------  Firamare ------------------------------------------  Sakandare ---------------------------------------  Gaba da sakandere ---------------------------  Wasu (kayyade) __________________ | 1  2  3  4  5 |  |
| A5 | Dadewan ka/ki a cikin wannan tsana’ar? | Kasa da shekara daya……………………  Shekara daya zuwa uku………………….  Sama da shekaru uku……………………. | 1  2  3 |  |

**SECTION B: KNOWLEDGE OF ZOONOTIC TB PREVENTION**

| QN | QUESTIONS & FILTERS | RESPONSE OPTIONS | CODE | SKIP TO |
| --- | --- | --- | --- | --- |
| B1 | Menene alamun cutan tarin fuka a dabbobi? (Kira biyu) |  |  |  |
| B2 | Cutan tarin fuka mai yaduwa kan kama dan adam? | i………………………………………………..  a’a…..…………………………………………  Ban sani ba………….……………………… | 1  2  3 |  |
| B3 | Cutan tarin fuka mai yaduwa kan yadu ta wace hanya? | Ci da shan kayan dabbobin da sun harbu da cutar (madara da nama)…………  Shan iska…..…………………………………  Ban sani ba……..……………………………  Wasu (kayyade)…………………………. | 1  2  3  4 |  |
| B4 | Menene alamun cutar tarin fuka a jikin dan adam?  (Kira biyu) |  |  |  |
| B5 | Za’a iya tsare cutar tarin fuka mai yaduwa daga shanu zuwa ga dan adam? | i……..………………………………………..  a’a……………………………………………  Ban sani ba………………………………… | 1  2  3 |  |
| B6 | Dafa madara kafin a sha kan kasha kwayoyin cutar tarin fuka mai yaduwa | i……..………………………………………..  a’a……………………………………………  Ban sani ba………………………………… | 1  2  3 |  |
| B7 | Raba dakin dabbobi daga dakin dan adam kan rage yaduwan cutar tarin fuka | i……..………………………………………..  a’a……………………………………………  Ban sani ba………………………………… | 1  2  3 |  |
| B8 | Za’a iya warkar da cutar tarin fuka mai yaduwa gabaki daya daga dan adam | i……..………………………………………..  a’a……………………………………………  Ban sani ba………………………………… | 1  2  3 |  |
| B9 | Wace irin jinya ne mafiyi da ta dace da cutar tarin fuka? | Maganin asibiti….……………………………  Maganin gargajiya………..………………..  Addu’a…………………………………………  Wasu (kayyade)…………………………….. | 1  2  3  4 |  |
| B10 | Jinyar cutar tarin fuka kyauta ne | i……..………………………………………..  a’a……………………………………………  Ban sani ba………………………………… | 1  2  3 |  |

**SECTION C: PRACTICES ABOUT ZOONOTIC TB PREVENTION**

| QN | QUESTIONS & FILTERS | RESPONSE OPTIONS | CODE | SKIP TO |
| --- | --- | --- | --- | --- |
| C1 | Ta yaya za’a kare yaduwar cutar tarin fuka daga shanakai? | Rigakafin BCG (Mai bincike kan duba ya/ta yadda)………………………..……………….  Jinya da kayi da kanka….…………………..  Amfani da maganin gargajiya………………  Addu’a………………………………………… | 1  2  3  4 |  |
| C2 | Ba na shan madara | i….………………………………………  a’a………………………………………. | 1  2 |  |
| C3 | Menene ka ke/ki ke yi in ka/ki lura cewa shanakan ka/ki sun harbu da cutan tarin fuka? | Sayar wa al’umma…………………………  A yanka kuma a binne shi…………………  A yanka a ci a gida…………………..……..  Wasu (kayyade)………………………. | 1  2  3  4 |  |
| C4 | Kullayomi ina kaidar da likitocin dabbobi a yanayin cutan shanakai ko naman su | i….………………………………………  a’a………………………………………. | 1  2 |  |
| C5 | Kullayomi ina yadda likitocin dabbobi su bincike dabbobi na | i….………………………………………  a’a………………………………………. | 1  2 |  |
| C6 | Kullayomi ina debi kashin dabbobi da sanya kayan kariya a hannu na | i….………………………………………  a’a………………………………………. | 1  2 |  |
| C7 | Bani zama tare da dabbobi a gida na | i….………………………………………  a’a………………………………………. | 1  2 |  |
| C8 | Menene zaka/zaki yi in ka/ki harbu da cutar tarin fuka mai yaduwa? | Nemi jinya a asibiti…………………………  Yi amfani da maganin gargajiya…………..  Nemi karewa daga iskoki…………………. | 1  2  3 |  |
| C9 | Ina ziyarar asibiti domin gwada lafiyan jiki akai akai | i….………………………………………  a’a………………………………………. | 1  2 |  |
| C10 | Kullayomi ina kare ciwo/gembu na in ina rike ko yin ma’ammala da dabbobi | i….………………………………………  a’a………………………………………. | 1  2 |  |
| C11 | Ba na cin abinci in ina rike ko yin mu’ammala da dabbobi | i….………………………………………  a’a………………………………………. | 1  2 |  |
| C12 | T yay aka ke/ki ke tsaftata kan ka/kan ki game da yaduwar cutar tarin fuka? Kira su |  |  |  |
